# O063: Healthcare-associated bloodstream infections in Finland, 1999-2011 – adjusted ranking of hospitals by Staphylococcus aureus rates

**DOI:** 10.1186/2047-2994-2-S1-O63

**Published:** 2013-06-20

**Authors:** T Kärki, J Ollgren, O Lyytikäinen

**Affiliations:** 1 National Institute for Health and Welfare, Helsinki, Finland

## Introduction

Bloodstream infections (BSI), including those caused by Staphylococcus aureus (SA-BSI) are often severe. Relatively large proportion of SA-BSIs are preventable, and their rate has been used as an indicator for hospital performance in infection control.

## Objectives

The objective of this study was to analyze the Finnish surveillance data in order to assess hospital rankings according to crude and adjusted rates of overall BSI and SA-BSI.

## Methods

11 Finnish hospitals conducted prospective incidence surveillance for healthcare-associated BSIs 1999-2011. A common protocol for laboratory-based case finding was used and only BSIs with onset >48 hrs after admission were included. Patient-days with specialties were obtained from hospitals' information technology departments to calculate incidence densities (ID) with 95% confidence intervals (CI). The ranking positions of hospitals were calculated for crude IDs and IDs adjusted by specialties and hospital type in mixed effect's negative binomial regression model. The effects in the model were considered to be constant over selected time period. The agreement and the correlation between rankings of the IDs were assessed by Cohen's kappa and Spearman's correlation coefficient, respectively.

## Results

We identified 7855 BSIs of which 990 were SA-BSIs. For all BSIs, IDs varied from 0.16 per 1,000 patient-days to 0.79 between hospitals and for SA-BSIs from 0.03 to 0.10. There were clear differences in crude and adjusted ranking positions of hospitals, but CIs were wide and mostly overlapped. The agreement between adjusted rankings was more fair than with crude ranking with its adjusted counterpart, kappa 0.3 (p=0.018) vs. 0.2 (p=0.078). Agreement of the two crude rankings was 0.1 (p=0.63). Correlation coefficients were 0.81 for adjusted BSI and SA-BSI rankings, and 0.69 for crude rankings.

## Conclusion

Both the overall BSI ranking and SA-BSI ranking identified outliers. Adjusting by specialties and hospital type may be needed when ranking overall BSI rates but not for SA-BSI rates. SA-BSIs can be a useful indicator for hospital performance, stimulating the use of surveillance data. However, the rankings must be interpreted with caution, especially when numbers are small during a short period of surveillance.

## Disclosure of interest

None declared.

